# A study on light transmittance through red protective shields modified with different window films

**DOI:** 10.1038/bdjopen.2017.14

**Published:** 2017-06-30

**Authors:** Vanida Nimmanon, Praewpat Pachimsawat, Siribang-on Pibooniyom Khovidhunkit, Bhornsawan Thanathornwong, Thirayost Nimmanon

**Affiliations:** 1 Department of Advanced General Dentistry, Faculty of Dentistry, Mahidol University, Bangkok, Thailand; 2 Department of General Dentistry, Faculty of Dentistry, Srinakharinwirot University, Bangkok, Thailand; 3 Department of Pathology, Phramongkutklao College of Medicine, Bangkok, Thailand

## Abstract

**Objectives/Aims:**

This study aimed to improve effectiveness of red protective shields in filtering unwanted light using window films.

**Materials and Methods:**

Red protective shields were modified by placing V-Kool (VK), Scotchtint (ST) or Hüper Optik (HP) window films on both sides. Percentage transmittance (%T) of light with a wavelength of 190–990 nm was determined using a double-beam ultraviolet (UV) and visible spectrophotometer.

**Results:**

In UV light (190–390 nm) and visible light (430–590 nm) ranges, %T in all modified groups and the control was below 2.5%. An increase in %T was observed at the wavelength of 630 nm, when all the modified shields showed superior effectiveness in light filtration over the control. In the infrared spectrum (700–990 nm), %T in the control was constantly high, ranging from 86 to 91%, compared to %T of 2–38% in all the modified groups, with the application of VK on both sides being the most effective group, followed by a combination of VK and HP.

**Conclusion:**

This study has introduced an economical and simple, yet highly effective, means of enhancing the efficiency of a red plastic protection shield in filtering unwanted infrared light, thereby additionally providing protection for dental personnel from potential ocular damages.

## Introduction

Nowadays, the light curing unit is an essential equipment in the dental clinic. It is available in many types, with the quartz–tungsten–halogen light curing unit being the most commonly used type in the dental office. This instrument employs a tungsten halogen lamp to generate radiation with wavelengths between 350 and 2,500 nm. These wavelengths include ultraviolet (UV), visible light and infrared spectra, with an emission peak between 970 and 980 nm.^1^ The radiation with wavelengths of less than 380 nm and more than 520 nm is undesired and thereby purged,^2,3^ leaving a broad spectral range of light emission for the use in the curing process of a composite resin.^4^ Light at a wavelength of 468 nm is the most effective in activating composite resins^5^ as well as other light-sensitive dental materials, such as bonding agents, glass ionomer cements and sealants.^6^ However, this blue visible light is not always safe to the eyes, given that it has been reported to cause eye injuries with possible association with development of age-related macular degeneration,^7^ a leading cause of permanent visual loss and blindness in aged people.^8^ Importantly, even though all four types of light curing units tested were not associated with a risk of ocular damages mediated by UV light, potential ocular hazards mediated by blue light were demonstrated upon cumulative eye exposure of the light generated by high-power lamps for only 6 s at a 30-cm distance over an 8-h working day.^3^ Furthermore, infrared light has been shown to be hazardous to both the lens^9^ and the retina,^10^ leading to the suggestion of it being a potential contributory factor of cataract and photothermal retinal injuries, respectively.

Many studies have reported that light curing units not only provide blue visible light, but also emit other unwanted spectra of light. An investigation on radiation emission from nine commercial light curing units showed that only two of them produced radiation at an acceptable wavelength, whereas many of the rest undesirably emitted light with wavelengths above the blue visible light spectrum, and two of these units also radiated light within the infrared spectrum.^11^ Supporting this previous investigation, only three out of five commercial light curing units were demonstrated to emit light within a suitable wavelength range.^12^ According to the threshold limit value of the American Conference of Governmental Industrial Hygienists, the maximum permissible exposure duration in 24 h was shown in 12 models of curing light units to range from 4 to 110 min, with the highest value resulting from an old unit.^13^ With regard to this finding, cumulative blue light retinal exposure over an 8-h working day as a result of a total of eight 30-s cures would exceed the threshold limit value recommended by the American Conference of Governmental Industrial Hygienists, provided that the light source was positioned where dentists could see the exposed tip. In addition, the authors noted the highly possible occurrence of lamp deterioration and filter cracking as a result of long-termed use of light curing units, suggesting the risk of eye injuries to dentists and their assistants if insufficiently protected. Despite the presence of well-established guidelines for eye safety in operative dentistry, ignorance and thereby suboptimal conformation to these rules among dental personnel have been reported.^14^

In order to provide safety and protection for the eyes from hazardous radiation generated by light curing units, light protective equipment has been developed. There are four major types of protective shields, including safety glasses, caps of light conductor tips or skirt shields, unit-attached eye shields, and unit-independent eye shields or separated shields.^15^ Protective shields are usually made of orange glasses,^16^ given that orange and blue are complementary colours, and as such, the orange colour efficiently absorbs blue visible light. Supporting this, orange protective shields were confirmed to have superior capability to eliminate blue visible light when compared to red protective shields.^12^ However, one study proposed the use of yellow or red protective shields, instead of orange ones, to prevent eye hazards.^17^ Our group have previously examined light transmittance through orange unit-independent protective shields, showing 2% light transmittance in the wavelength range of 200–500 nm (UV and visible light) and 80–90% light transmittance in the wavelength range of more than 500 nm, suggesting partial filtering efficiency of the shields for light with higher wavelengths.^18^ Importantly, supporting the use of red protective shields, our group have also investigated percentage light transmittance of locally made plastic shields of different colours, revealing that red shields more effectively filtered radiation than orange shields did, even though neither of them was able to filter infrared light.^19^

Given that none of the commercially available protective shields can provide complete filtration of unwanted light while allowing optimal operation view, this study aimed to improve filtration effectiveness of the shields by modifying them using window films. Red protective shields were covered on both sides with a combination of three types of window films: V-Kool (VK), Scotchtint (ST) and Hüper Optik (HP). Percentage transmittance (%T) of light in the wavelength range of 190–990 nm was determined using a double-beam UV and visible spectrophotometer.

## Materials and Methods

Red translucent plastic plates (Thai Poly Acrylic Corp, Bangkok, Thailand), 3 mm in thickness, were cut into 210 rectangular pieces with a dimension of 2 cm×1.3 cm using an electric cutting machine. These pieces of plastic shields were covered with a combination of three different types of window films, consisting of VK Vicole 70 (Southwall Technologies, Palo Alto, CA, USA), Scothtint RE 65NIARL (ST, 3M Solar Optical Products, St Paul, Minnesota, MN, USA) and Hüper Optik HP Sech (Southwall Technologies). They were divided into seven groups, including the control without modification, VK on both sides (2VK), ST on both sides (2ST), HP on both sides (2HP), a combination of VK and ST (VK+ST), a combination of ST and HP (ST+HP) and a combination of VK and HP (VK+HP). All groups were evaluated for %T at different wavelengths from 190 to 990 nm with 40 nm increments using a double-beam UV and visible spectrophotometer (Perkin-Elmer UV and Visible Spectrometer, Lambda 14). Each specimen was tested twice, and %T was shown as mean values. Statistical analysis was performed using ANOVA and Scheffe multiple comparison tests. Significance was assumed when *P*<0.05.

## Results

**Figure 1:**
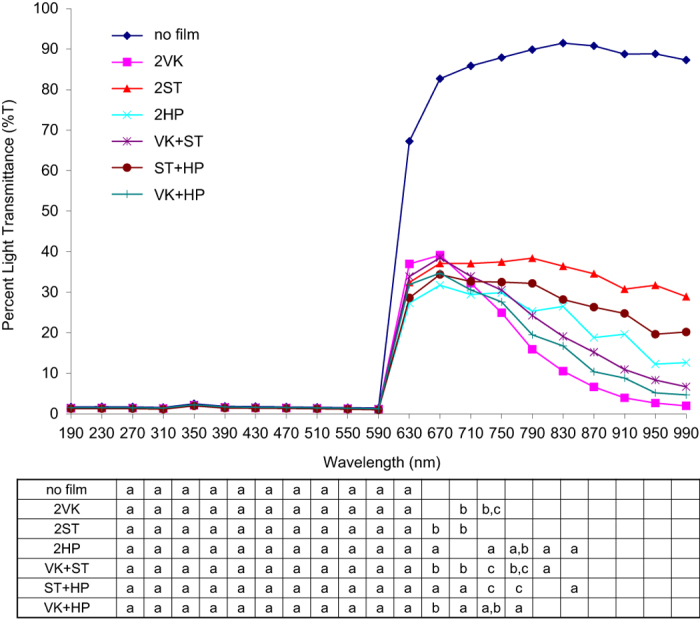
A comparison of percentage light transmittance of red translucent plastic shields with and without applications of different window films: VK, ST and HP at different wavelengths. The table demonstrates statistical analysis comparing between groups. The groups that are indicated by the same letters (a–c) at a wavelength are not statistically different (*P*>0.05) from each other.

%T was determined for red plastic shields with or without modifications at wavelengths between 190 and 990 nm. All data are shown in Table 1 and Figure 1. In the range of either UV light (190–390 nm) or visible light (430–590 nm), all groups where shown to have less than 2.5% light transmittance with no significant difference observed between the shields with and without modifications. In the range of 590–670 nm, light transmittance in the control group was dramatically increased to 67% and 83% at the wavelengths of 630 nm and 670 nm, respectively. In contrast, light transmittance in all the modified groups was increased to the maximum of 40% at both these wavelengths, which was significantly less than the control. Among the modified groups, 2HP was shown to have the lowest light transmittance in this wavelength range (630–670 nm), followed by ST+HP, 2ST, VK+HP, VK+ST and 2VK, respectively.

When the wavelengths reached the infrared range (710 nm), light transmittance in the control group remained constantly high, ranging from 86 to 91%, with a slight increase between 710 and 830 nm and a slight decrease between 830 and 990 nm. On the contrary, light transmittance in all the modified groups was gradually decreased until the wavelength of 990 nm and was shown to be significantly less than the control group throughout the range of 710–990 nm, with the 2ST group and the 2VK group being shown to have the highest and the lowest transmittance across the modified groups, respectively. The differences in light transmittance among the modified groups became more clearly evident at higher wavelengths, particularly those between 870 and 990 nm. In this extreme wavelength range of 870–990 nm, the lowest light transmittance was demonstrated in the 2VK group, followed by VK+HP, 2HP, VK+ST, ST+HP and 2ST groups, respectively.

## Discussion

Most studies on ocular damages caused by light generated by light curing units have focused on UV light^20^ and visible light,^7^ since these wavelength spectra are known for their causative relationship with retinal injuries.^21,22^ Accordingly, protective equipment has been designed to filter these light spectra to decrease exposure of dentistry personnel to these undesired wavelengths of light. An investigation demonstrated that all four types of light curing units, consisting of plasma arc, low-power light-emitting diode, high-power light-emitting diode and quartz–tungsten–halogen, radiated a negligible amount of effective UV light under all curing conditions and thereby did not increase the risk of UV light-induced ocular damages.^3^ Furthermore, many investigations have shown the effectiveness of protective shields in filtering UV light.^12,18,19^ Confirming this, we have shown in this present study that red plastic protective shields were able to filter UV light up to 98%, allowing only 2% of it transmitting through the shields. The highly efficient filtering capacity of these shields can be at least partly attributed to intrinsic properties of plastic material, which contains chemical bonds that absorb the energy of the radiation at a lower wavelength.

In contrast to the ability of the shield to filter UV light, the shields without modification was shown to be less effective in preventing transmittance of visible light (400–700 nm), which is composed of seven colours (violet, purple, blue, green, yellow, orange and red).^6,11^ Given that red is a colour complementary to green, this colour is theoretically highly effective in filtering green light but less effective in filtering red light. Noteworthy, our group have reported a superior filtering ability of red plastic shields over orange plastic shields, which are commonly used in dental practice.^19^ Importantly, the modifications of the shields using window films were shown to significantly reduce percentage transmittance of light in this visible range by at least 55%, thereby adding further protection against visible light-related eye injuries.

In contrast to UV light and visible light, the relationship between infrared light (700–990 nm) and ocular injuries is often overlooked. For example, a recent investigation evaluated protective filters against radiation specifically within the wavelength range of 400–525 nm,^23^ which belongs to the visible light spectrum. Nevertheless, biohazards of this infrared light has also been occasionally reported. One study demonstrated that proteins in the eye lens is notably sensitive to infrared radiation, suggesting the relationship between this light and development of cataract.^9^ Moreover, infrared light can also induce photomechanical, photothermal and photochemical damages to the retina,^10^ thereby posing a risk to visual impairment. In this present study, we considered a wider range of light, also including the infrared spectrum in the investigation. In contrast to the unmodified red plastic protective shields, which allowed up to 91% of infrared light to transmit through them, the modifications with window films were able to significantly reduce the transmittance of infrared light by 77–98% when compared to the unmodified shields.

Among all the three types of window films tested in this present study, the VK film was shown to be the most effective in light filtration. This VK film allowed some transmission of light within the harmless visible light spectrum, thereby providing adequate visibility for the dentist during an operation. Consistent with our previous investigation,^19^ up to 98% of infrared light was filtered when the VK film was applied on both sides of the shields. Furthermore, a combination of the VK film with another type of window films was shown to enhance light filtration effectiveness of the other window film compared to when it was used without the VK film, suggesting the complementary effect of the VK film to other film types. The infrared light filtrating capability of the VK film can be attributed to the silver particle in the metallic layer of the film, which has higher reflective properties than aluminium, nickel and copper, which are contained in the Scotchtinct film.^24^ It is not clear why the HP had less infrared light filtering capability than the VK film regardless of it also containing the silver particle in its metallic layer.

## Conclusion

There have been pieces of evidence showing that some light curing units that are generally used nowadays may emit some light in an inappropriate wavelength range,^11,12^ which could be hazardous to the eyes. We have demonstrated that unmodified plastic shields may not be able to provide adequate filtering for this undesired light, particularly that which is in the infrared spectrum. We therefore would like to recommend the application of window films, particularly the VK film, to the conventional protective shields in order to increase the filtration efficiency of the shields. This will further provide extra protection for the dentistry personnel from any light-induced ocular damages while allowing them to have sufficient visualisation during the operation.

## Figures and Tables

**Table 1:** A comparison of percentage light transmittance (%T) of red translucent plastic shields with and without a combination of window films

*Wavelength (nm)*	*No film*	*2 VK*	*2 ST*	*2 HP*	*VK+ST*	*ST+HP*	*VK+HP*
190	1.70±0.76	1.39±0.38	1.42±0.79	1.50±0.74	1.47±0.65	1.26±0.35	1.52±0.77
230	1.72±0.73	1.41±0.38	1.43±0.77	1.51±0.72	1.50±0.66	1.27±0.37	1.53±0.77
270	1.70±0.72	1.39±0.37	1.42±0.77	1.49±0.69	1.47±0.64	1.27±0.36	1.51±0.76
310	1.55±0.69	1.28±0.33	1.30±0.73	1.37±0.67	1.35±0.59	1.16±0.33	1.40±0.72
350	2.48±0.88	2.12±0.46	2.14±0.91	2.23±0.85	2.21±0.77	1.96±0.46	2.25±0.91
390	1.84±0.68	1.55±0.36	1.57±0.72	1.65±0.66	1.63±0.60	1.43±0.35	1.66±0.70
430	1.76±0.69	1.47±0.35	1.50±0.71	1.59±0.65	1.55±0.61	1.36±0.35	1.60±0.69
470	1.69±0.66	1.42±0.35	1.45±0.70	1.52±0.64	1.49±0.59	1.30±0.35	1.53±0.68
510	1.60±0.64	1.33±0.33	1.36±0.68	1.44±0.61	1.41±0.57	1.22±0.32	1.45±0.66
550	1.50±0.64	1.24±0.32	1.27±0.68	1.34±0.61	1.31±0.56	1.13±0.32	1.35±0.65
590	1.40±0.61	1.12±0.30	1.15±0.66	1.22±0.58	1.19±0.54	1.01±0.30	1.23±0.63
630	67.27±3.26	36.97±2.25	32.41±2.27	27.34±1.60	33.88±2.10	28.62±1.94	31.92±1.85
670	82.69±3.12	39.15±2.01	37.12±2.40	31.72±1.65	38.51±2.24	34.39±2.00	34.70±1.78
710	85.85±2.37	32.24±1.96	37.06±2.75	29.42±1.18	33.92±2.66	32.63±2.24	30.57±1.70
750	87.89±1.49	24.93±1.57	37.47±3.85	29.87±2.29	30.54±2.53	32.50±3.00	27.61±1.73
790	89.88±0.65	15.92±1.30	38.42±3.47	25.31±1.74	24.27±3.11	32.17±3.29	19.48±1.76
830	91.47±1.23	10.53±0.82	36.42±3.63	26.49±2.94	19.09±2.12	28.18±3.75	16.73±1.63
870	90.80±0.85	6.65±0.53	34.55±4.62	18.82±2.12	15.19±1.79	26.33±3.40	10.39±1.05
910	88.75±1.01	3.95±0.52	30.77±3.44	19.61±2.62	10.92±1.49	24.78±3.15	8.83±1.42
950	88.82±2.33	2.65±0.42	31.73±2.46	12.28±1.04	8.31±1.19	19.65±1.52	5.17±0.77
990	87.29±2.64	1.97±0.24	28.92±2.13	12.62±1.86	6.67±0.95	20.20±1.95	4.69±0.77

Abbreviations: HP, the Hüper Optik film; ST, the Scotchtint film; VK, the V-Kool film.
